# Human mobility variations in response to restriction policies during the COVID-19 pandemic: An analysis from the Virus Watch community cohort in England, UK

**DOI:** 10.3389/fpubh.2022.999521

**Published:** 2022-10-14

**Authors:** Tao Cheng, Tongxin Chen, Yunzhe Liu, Robert W. Aldridge, Vincent Nguyen, Andrew C. Hayward, Susan Michie

**Affiliations:** ^1^SpaceTimeLab for Big Data Analytics, Department of Civil, Environmental and Geomatic Engineering, University College London, London, United Kingdom; ^2^Institute of Health Informatics, University College London, London, United Kingdom; ^3^Institute of Epidemiology and Health Care, University College London, London, United Kingdom; ^4^Centre for Behaviour Change, University College London, London, United Kingdom

**Keywords:** COVID-19, GPS data analysis, pandemic, physical activity, human mobility, mobility inequality

## Abstract

**Objective:**

Since the outbreak of COVID-19, public health and social measures to contain its transmission (e.g., social distancing and lockdowns) have dramatically changed people's lives in rural and urban areas globally. To facilitate future management of the pandemic, it is important to understand how different socio-demographic groups adhere to such demands. This study aims to evaluate the influences of restriction policies on human mobility variations associated with socio-demographic groups in England, UK.

**Methods:**

Using mobile phone global positioning system (GPS) trajectory data, we measured variations in human mobility across socio-demographic groups during different restriction periods from Oct 14, 2020 to Sep 15, 2021. The six restriction periods which varied in degree of mobility restriction policies, denoted as “Three-tier Restriction,” “Second National Lockdown,” “Four-tier Restriction,” “Third National Lockdown,” “Steps out of Lockdown,” and “Post-restriction,” respectively. Individual human mobility was measured with respect to the time period people stayed at home, visited places outside the home, and traveled long distances. We compared these indicators across the six restriction periods and across socio-demographic groups.

**Results:**

All human mobility indicators significantly differed across the six restriction periods, and the influences of restriction policies on individual mobility behaviors are correlated with socio-demographic groups. In particular, influences relating to mobility behaviors are stronger in younger and low-income groups in the second and third national lockdowns.

**Conclusions:**

This study enhances our understanding of the influences of COVID-19 pandemic restriction policies on human mobility behaviors within different social groups in England. The findings can be usefully extended to support policy-making by investigating human mobility and differences in policy effects across not only age and income groups, but also across geographical regions.

## 1. Introduction

During this global pandemic, a variety of domestic and international restriction measures have been adopted by governments, such as stay-at-home (SAH) orders, national lockdowns, international travel bans, and public transportation closures (1, 2). As a result, the inequalities of human mobility behaviors caused by the pandemic mobility restrictions in different social groups are highlighted in related studies (3, 4). In other words, the abilities that afford the restriction measures, in terms of mobility behaviors, are differentiated by socio-demographic characteristics in human society. The human mobility inequality in socio-economic groups during the COVID-19 pandemic is essential for understanding the ability of fragile people to respond to restriction policies. So, in order to evaluate the social-health impact of the tremendous mobility shifting in society, it is useful to measure human mobility behavior changes in various populations in response to different restriction policies.

In this study, we aim to evaluate the effects of the COVID-19 restriction policies on mobility behaviors and their inequalities among different individuals in England. We utilize GPS tracker data as well as longitudinal individual and household surveys—both of which were sourced from the Virus Watch cohort study (5)—to fill the aforementioned research gaps. We developed an analysis framework to measure such inequalities by defining individual human mobility indicators and comparing their difference with respect to socio-demographic groups during the restriction periods. To the best of our knowledge, this study is the first longitudinal study using individual GPS trajectories to analyse the mobility behavior changes attributed to government containment interventions. Findings relating to mobility changes across socio-demographic groups may allow policymakers to develop informed and tailored COVID-19 interventions while pursuing social equity in future decision making.

The remainder of this paper is organized as follows. Section 2 reviews the related work of the human mobility variations and inequalities aided by mobile phone GPS data during the pandemic. Section 3 introduces our data resources and proposed analysis framework for detecting individual human mobility indicators. Section 4 illustrates a case study regarding the human mobility variations with respect to the restriction period and socio-demographic group. Finally, Section 5 discusses the implication of our findings, then concludes our contributions and reveals the study limitations and further research possibilities.

## 2. Reviews of related works

In the background of this unprecedented COVID-19 pandemic challenge, the tremendous reshaping of human mobility patterns in global cities, affected by all-encompassing restrictions, has aroused concern in research communities (6, 7). Empirically, various studies have reported that counties, regions, and cities had witnessed a sharp human mobility reduction and disruption, due to such restriction policies (8–11).

Though mobility restriction policies have been identified as efficient interventions in controlling the spread of infectious viruses in the population (12–16), such rapid social measures have been found to impose negative influences on public health and lifestyle during the COVID-19 crisis. Various types of human mobility behaviors are highly associated with physical and mental health benefits, such as physical activity (e.g., walking, cycling) (17–20). Notably, several public health outcomes caused by mobility activity restrictions have been reported during the period of COVID-19 response measures. It has been reported that unhealthy lifestyles (e.g., food consumption and meal patterns), increased daily sitting time, and decreased well-being are related to the restriction of physical activity (21–24). Mentally, stress, anxiety, frustration, and boredom have been identified as being associated with the limited physical activity during stay-at-home (or quarantine) periods (25, 26).

As human mobility decreased as a result of COVID-19 restriction polices, research has revealed that the rapid introduction of such social distancing limits may have an unequal influence on the everyday mobility behaviors of residents in the context of socio-economic disparities (27). Especially, income disparity has a high association with mobility variations in response to restriction policies. For example, Weill et al. (3) found that mobility reduction in affluent (high-income) and deprived (lower-income) areas showed significantly different impacts, as affected by emergency declarations in a state-level analysis in the US. Gauvin et al. (4) also suggested that unemployment, agriculture worker proportion, and high education level are strong determinants in explaining the mobility reduction during the pandemic, based on a province-level analysis in Italy. In addition, Hunter et al. (28) highlighted that walking, as a proxy of human mobility behavior of citizens from low-income areas, and high use of public transportation have had a greater impact on the COVID-19 emergency response.

Mobile phone GPS trajectory data have recently emerged as a kind of popular and valuable data source for human mobility and COVID-19 pandemic research (29). GPS data can provide an individual's location sequence information over a given time span, which contributes to almost real-time snapshots of human mobility, helping to overcome the limitations of self-reported surveys (30, 31). Using a combination of human mobility indicators generated from integrated mobile phone GPS data and urban data (e.g., points of interest, land use), researchers can investigate the effects of restrictions on dynamic human activity behaviors (e.g., commuting, place visits, travel mode) with fine granularity (32–34). Moreover, location-based mobile phone GPS data can be easily merged with census socio-demographic data by matching the geospatial information, which helps to examine the socio-economic determinants in human mobility variations.

Understanding the impact of mobility behaviors under different restriction policies and their inequality in the population can provide information for policy evaluation and devising related strategies to reduce the social-health impact of tremendous mobility shifting in society. However, considering the mixed socio-demographic characteristics of urban areas, area-level census or survey data are limited in ability to explain the inequalities of human mobility variations in the socio-demographic groups, due to insufficient individual-level information. In addition, previous studies have mainly focused on the effect of restriction in the initial period during pandemic outbreaks (i.e., the first national lockdowns, the first stay-at-home periods), thus lacking an evaluation of the effect of ongoing restriction or relaxation policies on human mobility and its inequality in socio-demographic groups.

In summary, the current literature suggests a comprehensive analysis consisting of elaborately measuring the human behavior variations and disentangling the inequality within diffident socio-demographic groups is required.

## 3. Materials and methods

### 3.1. Data resource and description

Table 1 summarizes the dataset used in this work. Our data mainly consist of Virus Watch survey data and mobile phone GPS trajectory data (see Sections 3.1.1, 3.1.2), Ordnance Survey POI data and UK geographical boundary data (see Section 3.1.4).

**Table 1:** A summary of data sources.

**Category**	**Used fields**	**Description**	**Data source**
Virus Watch survey data	gender, age, income	Individual and household information with demographic and socio-economic status.	Virus Watch cohort
Virus Watch mobile phone GPS data	longitude, latitude, datetime	Mobile phone GPS trajectory data are recorded from Esri Track App used by the Virus Watch participants.	Virus Watch cohort
Ordnance Survey POI data	9 types, longitude, latitude	Point of interest data in 9 types in England.	Ordnance Survey
UK geographical boundary data (local authority level)	local authority name, polygon geographical information	Local authority geographical boundary in the UK.	Office for National Statistics

#### 3.1.1. Virus Watch survey data

Virus Watch ^1^ is a longitudinal national household community cohort study of COVID-19, which continuously provides vital informing for government planning, public health, and NHS responses to the ongoing pandemic in the UK (5). Since 22 June 2020, this study has recruited more than 50,000 individuals from over 25,000 households across England and Wales. Briefly, the lead householder participating in the study is required to complete an online baseline survey and several follow-up weekly or monthly surveys for each member of their household. These surveys collected individual and household information covering a variety of topics, including but not limited to demographic and socio-economic status, medical conditions, COVID-19 contact history, and vaccination. Additionally, to monitor how people's travel behavior has changed throughout the duration of the study, all adult participants in the Virus Watch study were asked about optional consent to use a GPS tracking application (i.e., ArcGIS Tracker, powered by Esri), installed on their mobile phone.

Table 2 denotes the gender, age, and income groups of 1,094 users, according their types and levels; that is, gender groups with two types: female (646) and male (448); age groups with four levels: <35 (83), 35–49 (132), 50–64 (452), >64 (427); and income (pounds) groups with four levels: 0–24,999 (272), 25,000–49,999 (406), 50,000–74,999 (199), >74,999 (217). To clarify, we consider that age and income are crucial factors in relation to infectious rate and shielded level, which strongly impact of individuals' behaviors. Furthermore, we use the predefined groups in Virus Watch to categorize the age and income. For age groups, we use similar levels as Navaratnam et al. (35)'s work with a merged process due to small sample size below age 35. For income groups, we use the same income levels as Beale et al. (36)'s study.

**Table 2:** The user numbers of sociodemographic groups in the Virus Watch cohort.

**Age/Income**		**0–24,999**	**25,000–49,999**	**50,000–74,999**	**>74,999**	**Total**
<35	Male	4	11	5	14	83
	Female	10	23	11	5	
35–49	Male	9	10	12	18	132
	Female	12	28	21	22	
50–64	Male	27	60	33	48	452
	Female	78	103	51	52	
>64	Male	51	80	38	28	427
	Female	81	91	28	30	
Total		272	406	199	217	1,094

There are 1,094 of 1,828 users with socioeconomic and demographic information.

#### 3.1.2. GPS data

In this study, mobile phone GPS trajectory data were collected from 1,828 participants who had used the Track App among all the Virus Watch participants. The GPS coordinates transmitted from the tracker can be used to formulate tracing trajectories, facilitating in-depth mobility analysis. Detailed variable specifications and configuration of the GPS data from the tracker have been introduced by the Track App provider, Esri ^2^. As the data collection preference depends on the user's compliance and their time of entry, each user's active days are not equal, as they may use the Track App for different days and in different periods. Supplementary Figure S1 shows the distribution of active days for all users (1828) in our observation time period. Specifically, 42.5% of users (776 users) had below 10 active days and only 1.4% of users (25 users) had above 300 active days. Given that only 1,094 of the 1,828 participants provided their socio-economic and demographic information (i.e., gender, age, income), the case study in Section 4.2 uses the GPS data of these 1,094 users. Furthermore, all of these users are in England. Therefore, this is an analysis considering England and not the whole of the UK; notably, other nations (Wales and Scotland) had slightly different policies than those of England.

#### 3.1.3. UK restriction periods

According to the pandemic situation in the local communities, the COVID-19 restriction policies adopted by the England government were adjusted several times since the first national lockdown announced on Mar 23, 2020, following a series of restricted measures, such as stay at home, work from home, and closures of pubs, hospitality, school, and non-essential business. Our observation period (14 October, 2020 to 15 September, 2021) incorporates six restriction periods (337 days in total), separated by different national or local restriction policies ^3^ in England. In detail, the six restriction periods with days and observed users are shown as Table 3. The largest observed user numbers are found in “Third National lockdown' with 965 and the minimal observed user numbers is in ‘Post-restriction” with 153. To clarify, the total observed user frequency of six periods are 3,978 from 1,828 users as some users can repeatedly occur at some restriction periods. Such uneven distribution of observed objects in each restriction period is related to the difference of active days in users (see Supplementary Figure S1), we also output the user numbers classified by the active periods (from 1 to 6) in Supplementary Figure S2. In line with the distribution of users' active days, we only find 59 users (3.2 %) with six active periods (i.e., the user occurs in each period).

**Table 3:** The days and observed users in the six restriction periods.

**Restriction period**	**Start date**	**End date**	**Days**	**Observed user numbers**
Three-tier Restriction	14/10/2020	04/11/2020	22	815
Second National Lockdown	05/11/2020	04/12/2020	30	779
Four-tier Restriction	05/12/2020	03/01/2021	30	739
Third National Lockdown	04/01/2021	08/03/2021	64	965
Steps out of Lockdown	09/03/2021	19/07/2021	133	527
Post-restriction	20/07/2021	15/09/2021	58	153

#### 3.1.4. POI data

In this work, we delineate areas based on the local authority boundary (polygon) and its inner points of interest (points) in typology (the related method is introduced in Section 3.2.1). The POI data set of England was provided by Ordnance Survey ^4^ and the local authority boundary data of England was provided by the Office for National Statistics ^5^. Specifically, nine types of places (i.e., “Accommodation, eating, and drinking,” “Transport,” “Commercial services,” “Attractions,” “Sport and entertainment,” “Education and health,” “Public infrastructure,” “Manufacturing and production,” and “Retail”) were provided for 333 local authority areas in England.

### 3.2. Analysis framework

In this section, we first illustrate that our analysis framework on detecting human mobility indicators from GPS trajectory data (see Sections 3.2.1–3.2.3), and the main statistical test method—Kruskal Wallis H (KWH) test for testing the statistical differences of mobility indicators across predefined groups (see Section 3.2.4).

**Figure 1:**
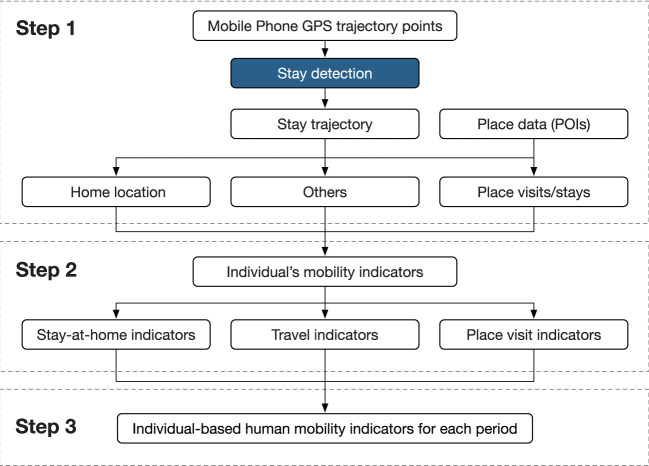
Analysis framework of detecting a user's daily mobility indicators from GPS trajectory data.

In detail, our analysis framework on detection processes of mobility indicators shown in Figure 1 is consists of three steps: Step 1 includes a stay detection algorithm, utilized to generate the stay trajectory from a user's daily mobile phone GPS trajectory points. Then, it extracts the user's home location, following which daytime place visits can be extracted based upon the detected stay trajectory. Step 2 defines and measure the human mobility indicators, incorporating the time duration and distance among stay-at-home and place visit. Step 3 calculates the individual-based mobility indicator for each observation period. These three parts are further explained in the following subsections.

### 3.2.1. Stay, home location, and place visit detection

**Figure 2:**
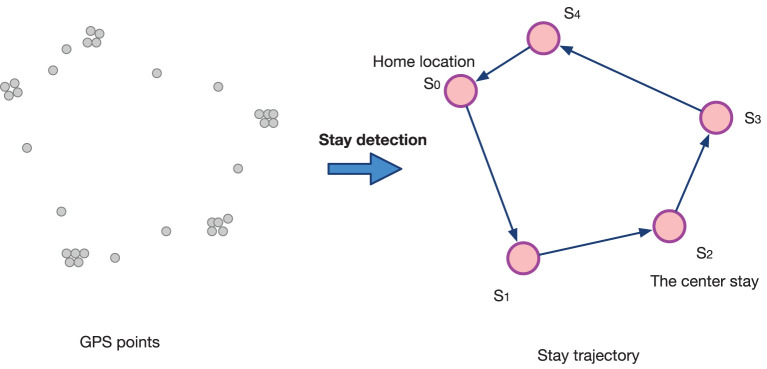
An example of a user's stay trajectory detected from raw GPS points.

A *stay* is a single user *u* spending some time at one place (i.e., the GPS point records of a user are at or around the same location during a time period) (37–39). Figure 2 shows the generation process of a user's stay trajectory from raw GPS points by stay detection. Specifically, a user's raw GPS points trajectory **P** can be denoted as a set of locations **l** with temporal information; then, each GPS point can be denoted as **P**_*i*_ = (**l**_*i*_, *t*_*i*_). As a stay trajectory **S** can be extracted from **P**_*i*_, each stay can be denoted as ${\text{S}}_{i}=\left({\text{l}}_{i},t_{i}^{\text{start}},t_{i}^{\text{end}}\right)$. In this analysis, we implement the stay detection algorithm proposed by Hariharan and Toyama (37), which requires two pre-defined parameters: Δ*d* (the maximum Euclidean distance that the points record a user's movement around a point location to count as a stay) and Δ*t* (the minimum duration that the GPS records stay within time distance to qualify as a stay at that location). In this work, Δ*d* and Δ*t* are defined as 2 min and 50 m, in order to delineate stays from raw GPS trajectory points.

Considering the semantic intuition in relation to human residence behavior and social activities at places, we can infer the user's home location and place visits in relation to the visit pattern from their detected stay trajectory (40–42). In this analysis, we define a user's (*u*) home location as the detected stay location that the user visits the most frequently during the night-time period. Home location detection *h* can be described as:


(1) $$\begin{array}{l} h\left(\text{S};u\right)=\arg\underset{i}{\max}|\left\{{\text{S}}_{i}|t_{i}^{\text{start}},t_{i}^{\text{end}}\in \left[t_{\text{nightbegin}},t_{\text{nightend}}\right]\right\}|. \end{array}$$

We define the night-time period to be from 10 pm to 6 am, in order to implement the home location detection (i.e., one user's home location is where a stay occurs the most times from 10 pm to 6 am for them).

Place visit detection aims to delineate a user's social activities at places (excluding their home location), which involves associating location information with each stay/stop (43). In this study, we use the region of interest (ROI) as the representation of place, which is combined with the related stay to describe a place visit through a heuristic procedure. First, we generate each ROI according to the topology of the point of interest (POI) using the overlapping area between its buffer zone (radius of 50 meters) and its Thiessen (Voronoi) polygon, which is a popular strategy used to define locations (e.g., retail store area) in geography (16, 44–46). Second, we extract the place visit by associating each stay with the ROI using a spatial connection.

### 3.2.2. Mobility indicators: Stay-at-home, travel, and place visit indicators

We define three indicators to represent individual mobility behaviors, based on the stay, home, and place visits detected above. In this study, human indicator detection aims to generate the representative indicators to characterize the stay-at-home, travel, and place visit behaviors from the detected individual's stay trajectory.

(1) Stay-at-home indicator. We define the stay-at-home indicator to be represented by the stay-at-home duration time (*hours*); that is, the total stay duration time at the home location. This duration time (which incorporates the night-time period) can be calculated from the detected home location stay on Section 3.2.1.

(2) Travel indicator. We define travel behaviors to be characterized by four indicators concerning the physical distance and home for a stay trajectory, including maximum distance from home, distance of mileage, maximum travel distance, and radius of gyration.

The maximum distance from home is the maximum value of the Euclidean distance between stays at the home location (47). For a user's stay trajectory **S**, the maximum distance from home *dh*_*max*_(**S**; *u*) is calculated as:


(2) $$\begin{array}{l} dh_{max}\left(\text{S};u\right)=\underset{1\leq i<n}{\max}|{\text{S}}_{i},h\left(\text{S};u\right)|, \end{array}$$

where |**S**_*i*_, *h*(*u*)| is the Euclidean distance (*km*) between a stay **S**_*i*_ and the home location *h*(**S**; *u*) (see equation 1), considering *n* stays. For example, the maximum distance from home in Figure 2 is the distance between the home location *S*_0_ and *S*_2_ (i.e., |*S*_0_, *S*_2_|).

The distance of mileage is the sum of the distance between two consecutive (time-ordered) stays (48, 49). For a user's stay trajectory **S**, the distance of mileage can be denoted as:


(3) $$\begin{array}{l} dm\left(\text{S};u\right)=\overset{n}{\underset{i=1}{\sum }}|{\text{S}}_{i-1},{\text{S}}_{i}|, \end{array}$$

where **S**_*i*−1_ and **S**_*i*_ are two successive stays in the *n* stays. In Figure 2, the distance of mileage can be calculated as *dm* = |*S*_0_, *S*_1_| + |*S*_1_, *S*_2_|+|*S*_2_, *S*_3_|+|*S*_3_, *S*_4_|+|*S*_4_, *S*_0_|.

The maximum travel distance is the maximum value of distance between two consecutive (time-ordered) stays (49). For a user's stay trajectory **S**, the maximum travel distance can be denoted as:


(4) $$\begin{array}{l} d_{max}\left(\text{S};u\right)=\underset{1\leq i<n}{\max}|{\text{S}}_{i-1},{\text{S}}_{i}|. \end{array}$$

For example, the maximum travel distance in Figure 2 is the distance between *S*_3_ and *S*_4_ (i.e., |*S*_3_, *S*_4_|).

The radius of gyration, as a radial distance to a point, is used to characterize the typical distance traveled by a center stay (time-ordered) in the mobility trajectory (50). For a user's stay trajectory **S**, the radius of gyration is defined as:


(5) $$\begin{array}{l} rg\left(\text{S};u\right)=\sqrt{\frac{1}{n}\overset{n}{\underset{i=1}{\sum }}{\left(\left|{\text{S}}_{i},{\text{S}}_{m}\right|\right)}^{2}}. \end{array}$$

For example, the radius of gyration in the trajectory in Figure 2 (the center stay is *S*_2_) can be calculated as $rg=\sqrt{\frac{1}{5}{\left(\left|{\text{S}}_{2},{\text{S}}_{0}\right|\right)}^{2}+{\left(\left|{\text{S}}_{2},{\text{S}}_{1}\right|\right)}^{2}+{\left(\left|{\text{S}}_{2},{\text{S}}_{3}\right|\right)}^{2}+{\left(\left|{\text{S}}_{2},{\text{S}}_{4}\right|\right)}^{2}}$.

(3) Place visit indicator. The place visit duration time is represented by the place visit duration time (*hours*) with respect to the nine types of places, which can be calculated as $dt(S_{i};u)=\left(t_{i}^{\text{end }}-t_{i}^{\text{start }}\right)$.

### 3.2.3. Indicators for each observation period

Therefore, our human mobility indicators are stay-at-home duration time, distance-based travel indicator (maximum distance from home, distance of mileage, maximum travel distance, radius of gyration), and place visit indicators are the visit duration times for nine types of places. As each restriction period had a different number of participants, we defined a mobility indicator for each restriction period, based on the individual mobility indicators defined above. This process followed two steps. First, for a user in one observation period, we calculate the mean of the human indicator values as the representative measure for the user in this observation period. Second, we use the mean of the mobility indicators of all users in this observation period as the mobility indicators for this period. These are defined as follows:

If there are *L* users in an observation period *T*_*k*_ with *M* days, we aim to generate an individual-based human mobility indicator $A^{T_{k}}$ as a representative measurement for *T*_*k*_. Suppose that a user *u, u* = 1, 2, 3, …, *L* has *N* active days in the observation period *T*_*k*_ (*N* ≤ *M*). As our temporal observation unit is daily, the human mobility indicator (e.g., maximum distance from home) for 1 day can be measured as $A_{u}^{d}$, *d* = 1, 2, 3, …, *N*. Then, the user's mobility indicator *A*_*u*_ in an observation period *T*_*k*_ can be denoted as


(6) $$\begin{array}{l} A_{u}^{T_{k}}=\frac{1}{N}\overset{N}{\underset{d}{\sum }}A_{u}^{d},d=1,2,3,\ldots ,N. \end{array}$$

So, the human mobility indicator $A^{T_{k}}$ for observation period *T*_*k*_ can be denoted as the mean of the mobility indicators for *L* users, which can be denoted as


(7) $$\begin{array}{l} A^{T_{k}}=\frac{1}{L}\overset{L}{\underset{u}{\sum }}A_{u}^{T_{k}},\;u=1,2,3,\ldots ,L. \end{array}$$

### 3.2.4. Statistical testing of the mobility indicator differences between groups

To examine the inequalities of mobility indicators between social groups, we select a statistical method called Kruskal-Wallis H (KWH) test to detect the differences of mobility indicators between groups with different socio-demographic characteristics (i.e., gender, age and income) or the groups of defined six restriction periods.

As a rank-based non-parametric test, the KWH test can determine if there are statistically significant differences of numerical variables between two or more groups of categorical variables with different sample sizes (51). Thus, the KWH test can be used to detect the mobility difference in the predefined groups with different sample sizes from our survey data, i.e., socio-demographic groups (shown as Table 2) and restriction period groups (shown as Table 3).

In the test process, the discrete distribution (*H* distribution) in KWH test assumes the *N* samples from *k* groups are identically shaped distributions. The *H* statistic can be denoted as:


(8) $$\begin{array}{l} H=(N-1)\frac{\sum_{i=l}^{k}n_{i}{\left({\bar{r}}_{i\cdot }-\bar{r}\right)}^{2}}{\sum_{i=l}^{k}\sum_{j=l}^{n_{i}}{\left(r_{ij}-\bar{r}\right)}^{2}} \end{array}$$

where, *n*_*i*_ is the number of samples in group *i*, *r*_*ij*_ is the rank of sample j (in all *N* samples) from group *i*, ${\bar{r}}_{i\cdot }=\frac{\overset{n_{i}}{\underset{j=1}{\sum }}r_{ij}}{n_{i}}$ is the average rank of all samples from group *i* and $\bar{r}=\frac{1}{2}\left(N+1\right)$ is the average of all the ranks (*r*_*ij*_). Then the critical statistic $\chi_{c}^{2}$ of *H* can be computed by the χ^2^ distribution approximation with *k*−1 degrees of freedom. We reject the null hypothesis (no difference between groups) if *H* is significant ($H>\chi_{c}^{2}$) and output the *p*-value.

## 4. Results

In the case study, we first generate six human mobility indicators for each individuals in our samples, then we summarized them into three socio-demographic types (age, gender and income) in six restriction periods, respectively, named as “Three-tier restriction,” “Second national Lockdown,” “Four-tier restriction,” “Third national lockdown,” “Steps out of lockdown,” and “Post-restriction.”

### 4.1. Impacts of restriction policies on mobility

*Stay-at-home and travel variations*. Table 4 shows the statistical description of individual's stay-at-home and travel indicators during the whole observation period. As the travel indicators (*km*) measure the travel behavior in distance, the maximum distance from home presents a maximum value of 193.28 *km*, indicating a long-distance journey across the main England area in 1 day. To the contrary, the minimum value (0 km) of the travel indicator indicates that a user has no movement (e.g., stayed at home) in one active day. Similarly, the maximum stay-at-home duration time (23.13 *h*) also denotes a user staying at their home location for a nearly whole day without distinctive travel.

**Table 4:** Statistical descriptions of individual's stay-at-home and travel indicators during the whole observation period.

**Human mobility indicators**	**Mean**	**Std**	**Min**	**Medium**	**Max**
Stay-at-home duration time (hour)	3.98	2.91	0.10	3.51	23.13
Distance of mileage (km)	13.62	18.87	0.00	8.31	324.99
Maximum distance from home (km)	6.84	10.77	0.00^*^	3.96	193.28
Distance of mileage (km)	13.62	18.87	0.00	8.31	324.99
Maximum travel distance (km)	6.89	9.56	0.00	4.19	137.82
Radius of gyration (km)	2.71	4.31	0.00	1.54	73.46

^*^ 0 km indicates a user has no movement (i.e., staying at home) in one active day during the observation period.

**Figure 3:**
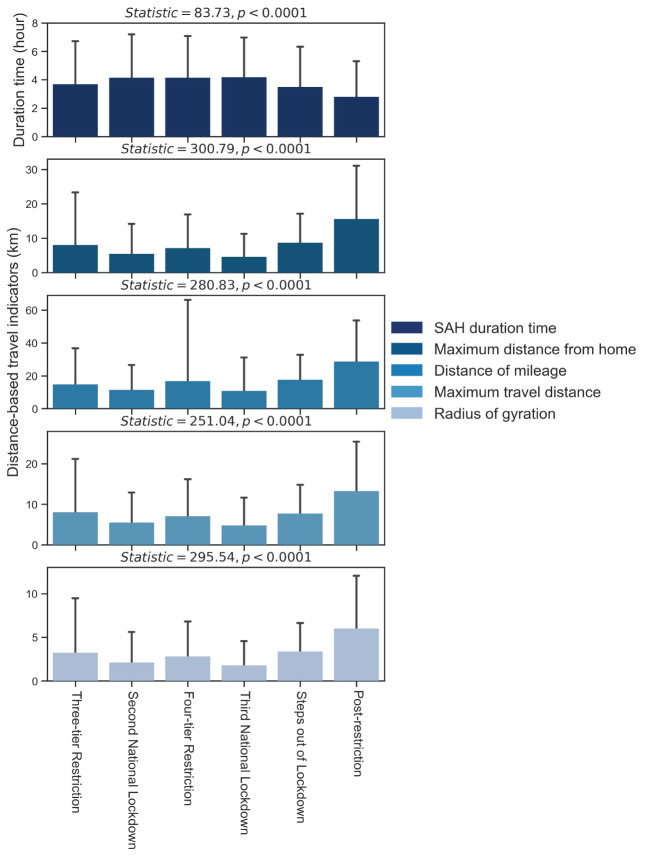
Stay-at-home and travel indicators variations (mean and standard deviation) in the different policy restriction periods.

In terms of the impact of restriction periods on stay-at-home and travel behaviors, Figure 3 indicates the variations in the stay-at-home and travel indicators, as well as the results of the Kruskal–Wallis H (KWH) test regarding the difference of each indicator in different restriction periods. The travel indicators (maximum distance from home, distance of mileage, maximum travel distance, and radius of gyration) in the second national lockdown and the third national lockdown are presented the lowest mean values, as related to the lockdown measures, such as the stay-at-home order ^6^ and work from home/school closure measures ^7^. The travel indicators started to increase in the steps out of lockdown period, with schools and colleges reopening due to relaxation policies ^8^. In addition, the stay-at-home duration time showed variations, with an increase while moving into the second national lockdown and a decrease in the steps out of lockdown period; however, it did not show a significant shift when going into the four-tier restriction period, as work from home measures remained valid in England ^9^.

We also observe that all indicators are statistically significant in different restriction periods. And further paired KWH tests of stay-at-home and travel indicators in different restriction periods are presented as Supplementary Figure S3. Though the majority of differences of mobility indicators between each two periods are statistically significant, some indicators of specific periods present no difference, such as the stay-at-home duration time between “Second National Lockdown” and “Third National Lockdown,” the maximum distance from home between “Three-tier Restriction” and “Four-tier Restriction.”

*Place visit variations*. To describe the place visit changes during restriction periods, we detected 1,479,079 stays and classified them to place visits and others using POI data in England. There are 769,843 (52 %) stays can be linked with the nine types of place information during the whole period. The detailed descriptions of detected stays and place visits in six restriction periods are presented in Supplementary Table S1.

Table 5 shows a statistical description of the visit duration time of individuals at places throughout the whole observation period. The mean level of visit duration time at each type of place is below 40 *mins*, while the max value of visit duration time is above 8 h, indicating some visit behaviors related to working at the workplace. In this regard, there is a minor part (only 19 of all 3,978 observed user frequency) of individuals' place visit duration time for a period (defined in Section 3.2.3) above 8 h. Then, nine types of place visits are all found statistically significant in six periods from the KWH test results (see Supplementary Figure S4). Further, the pair KWH tests of place visit indicators between each periods can be found at Supplementary Figure S5.

**Table 5:** Statistical description of individual's visit duration time at places during the whole observation period.

**Visiting places duration time (hour)**	**Mean**	**Std**	**Min**	**Medium**	**Max**
Accommodation, eating and drinking	0.37	0.53	0.03	0.22	6.26
Transport	0.35	0.61	0.03	0.15	9.65^*^
Commercial services	0.58	0.83	0.03	0.28	11.92^*^
Attractions	0.28	0.53	0.03	0.14	6.82
Sport and entertainment	0.52	0.87	0.03	0.24	10.90^*^
Education and health	0.52	0.79	0.03	0.22	6.61
Public infrastructure	0.33	0.58	0.03	0.15	7.41
Manufacturing and production	0.35	0.65	0.03	0.14	6.25
Retail	0.32	0.51	0.03	0.20	8.70

^*^ Visiting at some places above 8 h may be related to working behaviors.

To highlight the sequential change pattern, we use change rate to depict the variations of place visit indicators during restriction periods. The change rate are the change range of indicator value in the current period, compared to the former one, which can be denoted as:


(9) $$\begin{array}{l} Change\;rate=(Indicator_{current\;period}-Indicator_{former\;period})\;\\ \;/(Indicator_{former\;period}) \end{array}$$

**Figure 4:**
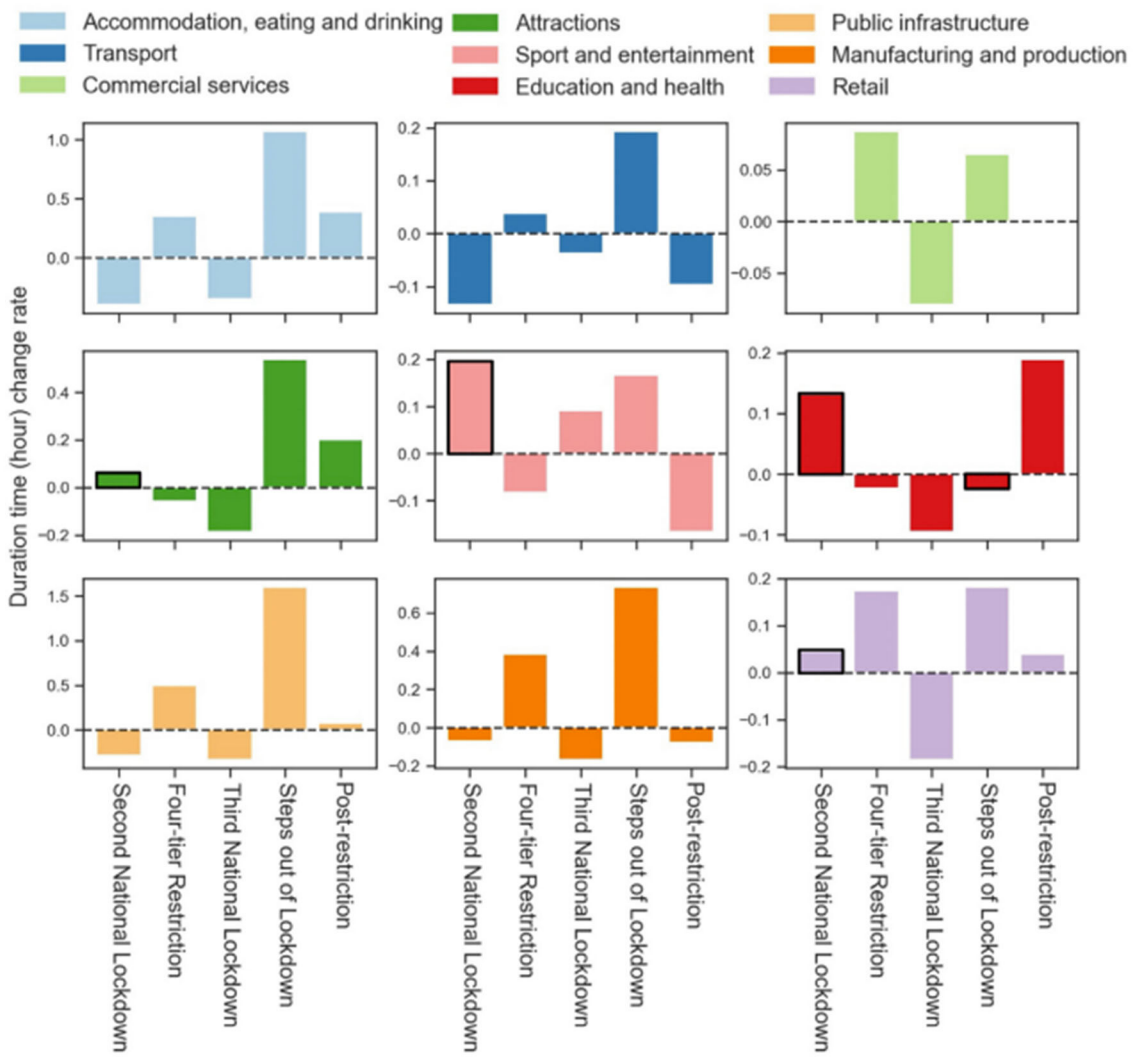
The change rates of place visit indicators in the six restriction periods. The bars with black boxes denote: (1) The increased change rates in “Second National Lockdown” (compared to “Three-tier Restriction”), and the declined change rate in “Step out of Lockdown” (compared to “Third National Lockdown”); (2) The KWH results show visit indicators are significantly different between the two consecutive periods.

Figure 4 shows the change rate of place visit time duration in different restriction periods. We find that the visit duration times at some types of places are not influenced by the restriction or relaxation policies. First, unlike the duration time at other places with reductions while going into the second national lockdown, the people staying at distinctive places (highlighted by black box) longer compared to the three-tier restriction period, such as attractions (change rate, 5%), sport and entertainment (change rate, 20%), education and health (change rate, 13%), and retail locations (change rate, 3%). These four types of place visit indicators between the second national lockdown and three-tier restriction period are all statistically significant differences (see Supplementary Figure S5). Second, only the visit duration time at sport and entertainment locations increased during the third national lockdown (change rate, 9%), compared to the four-tier restriction. However, the paired KWH test results show no significance between the two periods (see Supplementary Figure S5). Third, during the steps out of lockdown period, with a series of restriction measures lifting, only the duration time of visit at education and health locations (highlighted by black box) showed a slight reduction (change rate, 2%). The paired KWH test results also show a significant difference between the indicators of the two periods (see Supplementary Figure S5).

### 4.2. Human mobility variations by socio-demographic groups

In this part, to interpret the inequality in mobility, we examine the variations of mobility indicators (represented by stay-at-home and travel indicators) by socio-demographic groups. The mean value of stay-at-home and travel indicators, as well as their difference with respect to the three socio-demographic groups (gender, age, income) during the whole observation period are shown in Supplementary Tables S2–S4. It denotes the human mobility indicators with no difference in gender groups, and the stay-at-home indicators with no difference between age or income groups. On the contrary, the travel indicators showed significant differences in the two groups by the KWH test. Specifically, in Supplementary Table S3, the “ <35” age group's distance of mileage (mean value) presents the highest level compared to other age groups, while the maximum distance from home value in this group was lower than that of the “35–49” and “50–64” groups. In addition, Supplementary Table S3 indicates that the travel indicators of the lowest income group (0–24,999) presented lower values than other income groups, with the longest stay-at-home duration time during the whole period observed in this group. Then, all mobility indicators are significantly different in all restrictions periods, except the SAH duration time of <35 (in age groups) and 50,000–74,999 (in income groups) without statistically significant difference in all restriction periods (see Supplementary Figure S6).

**Figure 5:**
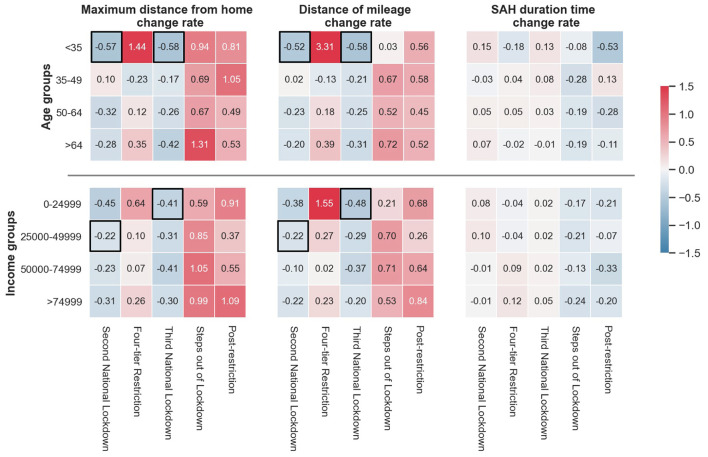
The change rates of mobility indicators in age and income groups during the six restriction periods. The red grids denote increases in change rate (> 0), and the blue ones represent reductions in change rate (< 0), with colour saturation indicating the change rate level. The black boxes highlight the grids for (1) The biggest change rates (absolute values) in “Second National Lockdown” and “Third National Lockdown”; (2) The KWH results show the indicators are significantly different between the current period and the former one.

To delineate the different shiftings of restrictions on mobility indicators in different socio-economic groups, we calculated the change rates (see Equation 9) of the human mobility indicators (maximum distance from home, distance of mileage and stay-at-home duration time) with respect to the age and income groups, as shown in Figure 5.

We observe the mobility indicator changes imposed by restriction policies in low-age and low-income groups are more fragile (change more) than those of other groups in our samples. In examining the influence of the second national lockdown and the third national lockdown, the maximum distance from home and the distance of mileage in the low-age (<35) and low-income (0–24,999) groups present the highest level of reduction (i.e., the biggest absolute change rate values) than other groups. In this regard, the black boxes highlight the change rate grids with the biggest absolute values (in “Second National Lockdown” and “Third National Lockdown”) and the significantly different mobility indicators between the current period and the former (see Supplementary Figures S7, S8) for detailed paired KWH test results of age and income subgroups). To emphasize, in terms of the difference of travel indicators in income groups between “Three-tier Restriction” and “Second National Lockdown,” only 0–24,999 income group (the grids with the black boxes in Figure 5) shows a statically significant between these two periods (see Supplementary Figures S7, S8).

Then, the stay-at-home duration times affected by the two national lockdowns are higher in the low-age group compared to other groups. In addition, we find that relaxation policies affected the low-age and -income groups more than other groups; for example, the maximum distance from home and distance of mileage of the “ <35” group in the four-tier restriction period increased by 144 and 331%, respectively. Meanwhile, the change rates of the maximum distance from home and the distance of mileage in the low-income (0–24,999) group in the four-tier restriction period were 64 and 155%, respectively.

## 5. Discussion and conclusions

In this study, we proposed an approach to estimate and compare the impacts of different restrictions on human mobility by generating individual human mobility indicators using mobile phone GPS data from the Virus Watch cohort in England, UK. Following our proposed analysis, we generated three aspects of human mobility behaviors, consisting of stay-at-home behavior (measured by duration time), travel behaviors (measured by the maximum distance from home, distance of mileage, maximum travel distance, and radius of gyration), and place visit behaviors (measured by the visit duration time in places), which presented statistically significant shifts between the different periods coinciding with the announced restriction policies.

Concerning the influences of restriction policies, the findings demonstrate that human mobility behaviors significantly differentiate across periods, from three-tier restrictions to the post-restriction period, in England. Further, the heterogeneity of human mobility indicator variations imposed by the restriction policies should be highlighted. For example, during the second national lockdown, with tougher restrictions, the length of times people visited attractions, sport and entertainment, education and health, and retail locations were longer than that in the tier-three restriction period. As a part of still-open essential facilities, human activities at these places were significantly influenced by the second national lockdown policy, with the exemption of stay-at-home orders for activities such as education, exercise, outdoor recreation, and shopping for food and essentials. We also noticed that, in the steps out of lockdown period, only the visit duration at education and health locations declined, which is not in alignment with the relaxation policy, as the re-opening of primary and secondary schools was announced on March 8, 2021. An alternative explanation for this is sample bias in our cohort community, as the age group below 35 was small, and students may not be well-represented in the Virus Watch app user group.

Considering the human mobility variation inequality concerning socio-demographic groups, the low-age and -income groups were more affected by restriction or relaxation policies. We also provided evidence that low-income and low-age groups were lower-activity and were more significantly impacted than other socio-economic groups during restriction periods. In particular, the travel indicator variations of these two groups during the second and the third national lockdowns demonstrated the lower ability of these two groups, compared to other groups, to adapt to such restrictions. Such mobility inequalities in the low-age and low-income groups are prominently related to the dependency on public transport facilities. In the context of England lockdowns, the low-age and low-income people can't travel a longer distance than normal times as the restricted bus, tube and train usage.

In addition, some limitations are highlighted as follows. As the different objects in different periods caused by the inconsistent activity days of users during the periods in the Virus Watch cohort, the estimations of human mobility indicators may yield biased results in terms of uneven distributions of socio-demographic group samples. Thus, the comparison of periods with different number of subjects reveals a comparison among different persons, and consequently, is not strong enough to assume this was caused by restrictions. In other words, the differences among periods could be a result of different subjects included in the different periods and not for the different restriction periods. In parallel, though the KWH test are not restricted by the different samples between groups, the non-adjustment in socio-demographic group size also can lead to the finding basis, especially the direct results related to the small samples in young people (i.e., <35 group).

In conclusion, this research estimated the impacts of different restriction periods on human mobility, using mobile phone GPS trajectories, in order to identify the mobility inequality between socio-economic groups during the COVID-19 pandemic. The results suggested all human mobility indicators significantly differed during the six restriction periods and the inequality in socio-demographic groups in England. The influences relating to mobility behaviors seems more substantial in younger and low-income groups than in other groups in the high-degree restriction policies in mobility (the second and third national lockdowns).

These results enhance our understanding of how restriction policies affect human mobility behaviors (e.g., stay-at-home, travel, and place visits) within social groups in England. Our findings are based upon data in England, but we believe this may apply to other countries given the young and low income groups are very similar at least in the Europe. The analytical framework of human mobility inequality based upon mobile GPS data can be adopted as an approach to disentangle social inequality for other areas during and after the pandemic. Disaggregating the results presented here could identify the inequality of human mobility in relation to low age and low income during the pandemic. Unlike area-level analysis in human mobility inequality, exploring these individual-level analytics might help to develop tailored and human-centric strategies considering specific social identities rather than simply areas.

Further research is required to retrieve and validate the human mobility indicators from large amounts of participants with consistent active days to reduce the observation sample bias. Then, it also needs a detailed estimation of human mobility inequality concerning various social activities (e.g., working, commuting and leisure), which could further reveal the resilience and recovery of mobility in local communities during the post COVID-19 pandemic.

## Data availability statement

The raw data supporting the conclusions of this article will be made available by the authors, without undue reservation.

## Ethics statement

Written informed consent was obtained from the individual(s) for the publication of any potentially identifiable images or data included in this article. Virus Watch is a national study that has been approved by the Hampstead NHS Health Research Authority Ethics Committee. Ethics approval number — 20/HRA/2320.

## Author contributions

TaC designed the study, participated in data analysis, and manuscript writing and revision. ToC participated in method design, data analysis, data visualization, and manuscript writing and revision. YL participated in data cleaning, data analysis, and manuscript writing. AH, SM, RA, and VN provided critical comments and participated in manuscript revision. All authors contributed to the article and approved the submitted version.

## Funding

The research is supported by the MRC Grant Ref: MC PC 19,070 awarded to UCL on 30 March 2020 and MRC Grant Ref: MR/V028375/1 awarded on 17 August 2020.

## Conflict of interest

The authors declare that the research was conducted in the absence of any commercial or financial relationships that could be construed as a potential conflict of interest.

## Publisher's note

All claims expressed in this article are solely those of the authors and do not necessarily represent those of their affiliated organizations, or those of the publisher, the editors and the reviewers. Any product that may be evaluated in this article, or claim that may be made by its manufacturer, is not guaranteed or endorsed by the publisher.

## Footnotes

^1^Virus Watch: https://ucl-virus-watch.net/.

^2^ArcGIS Tracker: https://www.esri.com/en-us/arcgis/products/arcgis-tracker/resources.

^3^Timeline of UK government coronavirus lockdowns and restrictions. https://www.instituteforgovernment.org.uk/charts/uk-government-coronavirus-lockdowns.

^4^Ordnance Survey: https://www.ordnancesurvey.co.uk/.

^5^Office for National Statistics: https://www.ons.gov.uk/.

^6^Prime Minister announces new national restrictions: https://www.gov.uk/government/news/prime-minister-announces-new-national-restrictions.

^7^Prime Minister's address to the nation: https://www.gov.uk/government/speeches/prime-ministers-address-to-the-nation-4-january-2021.

^8^Schools and colleges to reopen: https://www.gov.uk/government/news/schools-and-colleges-to-reopen-from-tomorrow-as-part-of-step-one-of-the-roadmap.

^9^Winter plan: https://www.gov.uk/government/publications/covid-19-winter-plan/covid-19-winter-plan.

## References

[1] Aktay A Bavadekar S Cossoul G Davis J Desfontaines D Fabrikant A. Google COVID-19 community mobility reports: anonymization process description (version 1.1). arXiv preprint arXiv:200404145. (2020) 10.48550/arXiv.2004.04145

[2] Hale T Angrist N Goldszmidt R Kira B Patherick A Phillips T. A global panel database of pandemic policies (Oxford COVID-19 Government Response Tracker). Nat Human Behav. (2021) 5:529–38. 10.1038/s41562-021-01079-8 33686204

[3] Weill JA Stigler M Deschenes O Springborn MR. Social distancing responses to COVID-19 emergency declarations strongly differentiated by income. Proc Nat Acad Sci USA. (2020) 117:19658–60. 10.1073/pnas.2009412117 32727905 PMC7443940

[4] Gauvin L Bajardi P Pepe E Lake B Privitera F Tizzoni M. Socio-economic determinants of mobility responses during the first wave of COVID-19 in Italy: from provinces to neighbourhoods. J R SocInterface. (2021) 18:20210092. 10.1098/rsif.2021.0092 34343450 PMC8331235

[5] Hayward A Fragaszy E Kovar J Nguyen V Beale S Byrne T. Risk factors, symptom reporting, healthcare-seeking behaviour and adherence to public health guidance: protocol for Virus Watch, a prospective community cohort study. BMJ Open. (2021) 11:e048042. 10.1136/bmjopen-2020-048042 34162651 PMC8230990

[6] Jia JS Lu X Yuan Y Xu G Jia J Christakis NA. Population flow drives spatio-temporal distribution of COVID-19 in China. Nature. (2020) 582:389–94. 10.1038/s41586-020-2284-y 32349120

[7] Kaur S Bherwani H Gulia S Vijay R Kumar R. Understanding COVID-19 transmission, health impacts and mitigation: timely social distancing is the key. Environ Dev Sustain. (2021) 23:6681–97. 10.1007/s10668-020-00884-x 32837280 PMC7368631

[8] Galeazzi A Cinelli M Bonaccorsi G Pierri F Schmidt AL Scala A. Human mobility in response to COVID-19 in France, Italy and UK. Sci Rep. (2021) 11:1–10. 10.1038/s41598-021-92399-2 34162933 PMC8222274

[9] Alfano V Ercolano S. The efficacy of lockdown against COVID-19: a cross-country panel analysis. Appl Health Econ Health Policy. (2020) 18:509–17. 10.1007/s40258-020-00596-3 32495067 PMC7268966

[10] Balmford B Annan JD Hargreaves JC Altoè M Bateman IJ. Cross-country comparisons of Covid-19: policy, politics and the price of life. Environ Resour Econ. (2020) 76:525–51. 10.1007/s10640-020-00466-5 32836862 PMC7400753

[11] Huang X Lu J Gao S Wang S Liu Z Wei H. Staying at home is a privilege: evidence from fine-grained mobile phone location data in the United States during the COVID-19 pandemic. Ann Am Assoc Geogr. (2022) 112:286–305. 10.1080/24694452.2021.1904819

[12] Wang S Liu Y Hu T. Examining the change of human mobility adherent to social restriction policies and its effect on COVID-19 cases in Australia. Int J Environ Res Public Health. (2020) 17:7930. 10.3390/ijerph17217930 33137958 PMC7662641

[13] Yabe T Tsubouchi K Fujiwara N Wada T Sekimoto Y Ukkusuri SV. Non-compulsory measures sufficiently reduced human mobility in Tokyo during the COVID-19 epidemic. Sci Rep. (2020) 10:1–9. 10.1038/s41598-020-75033-5 33093497 PMC7581808

[14] Kucharski AJ Russell TW Diamond C Liu Y Edmunds J Funk S. Early dynamics of transmission and control of COVID-19: a mathematical modelling study. Lancet Infect Dis. (2020) 20:553–58. 10.1016/S1473-3099(20)30144-4 32171059 PMC7158569

[15] Moosa IA. The effectiveness of social distancing in containing Covid-19. Appl Econ. (2020) 52:6292–305. 10.1080/00036846.2020.178906134894622

[16] Lai S Ruktanonchai NW Zhou L Prosper O Luo W Floyd JR. Effect of non-pharmaceutical interventions to contain COVID-19 in China. Nature. (2020) 585:410–3. 10.1038/s41586-020-2293-x 32365354 PMC7116778

[17] Guthold R Stevens GA Riley LM Bull FC. Global trends in insufficient physical activity among adolescents: a pooled analysis of 298 population-based surveys with 1·6 million participants. Lancet Child Adolescent Health. (2020) 4:23–35. 10.1016/S2352-4642(19)30323-2 31761562 PMC6919336

[18] Paul P Carlson SA Carroll DD Berrigan D Fulton JE. Walking for transportation and leisure among US adults–National Health Interview Survey 2010. J Phys Activity Health. (2015) 12:S62. 10.1123/jpah.2013-0519 25133651 PMC4582654

[19] Schiller JS Lucas JW Peregoy JA. Summary health statistics for US adults. In: National Health Interview Survey. Washington, DC (2012). 25116400

[20] Newman P Matan A. Human mobility and human health. Curr Opin Environ Sustain. (2012) 4:420–6. 10.1016/j.cosust.2012.08.005

[21] Ammar A Brach M Trabelsi K Chtourou H Boukhris O Masmoudi L. Effects of COVID-19 home confinement on eating behaviour and physical activity: results of the ECLB-COVID19 international online survey. Nutrients. (2020) 12:1583. 10.1159/000512852 32481594 PMC7352706

[22] Lesser IA Nienhuis CP. The impact of COVID-19 on physical activity behavior and well-being of Canadians. Int J Environ Res Public Health. (2020) 17:3899. 10.3390/ijerph17113899 32486380 PMC7312579

[23] Diniz TA Christofaro DG Tebar WR Cucato GG Botero JP Correia MA. Reduction of physical activity levels during the COVID-19 pandemic might negatively disturb sleep pattern. Front Psychol. (2020) 11:586157. 10.3389/fpsyg.2020.586157 33424702 PMC7793775

[24] López-Valenciano A Suárez-Iglesias D Sanchez-Lastra MA Ayán C. Impact of COVID-19 pandemic on university students' physical activity levels: an early systematic review. Front Psychol. (2021) 11:62456. 10.3389/fpsyg.2020.624567 33519653 PMC7845570

[25] Brooks SK Webster RK Smith LE Woodland L Wessely S Greenberg N. The psychological impact of quarantine and how to reduce it: rapid review of the evidence. Lancet. (2020) 395:912–920. 10.1016/S0140-6736(20)30460-8 32112714 PMC7158942

[26] Pfefferbaum B North CS. Mental health and the Covid-19 pandemic. N Engl J Med. (2020) 383:510–2. 10.1056/NEJMp2008017 32283003

[27] Zhang A Shi W Tong C Zhu X Liu Y Liu Z. The fine-scale associations between socioeconomic status, density, functionality, and spread of COVID-19 within a high-density city. BMC Infect Dis. (2022) 22:1–22. 10.1186/s12879-022-07274-w 35313829 PMC8936044

[28] Hunter RF Garcia L de Sa TH Zapata-Diomedi B Millett C Woodcock J. Effect of COVID-19 response policies on walking behavior in US cities. Nat Commun. (2021) 12:1–9. 10.1038/s41467-021-23937-9 34135325 PMC8209100

[29] Oliver N Lepri B Sterly H Lambiotte R Deletaille S De Nadai M. Mobile phone data for informing public health actions across the COVID-19 pandemic life cycle. Sci Adv. (2020) 6:eabc0764. 10.1126/sciadv.abc0764 32548274 PMC7274807

[30] Jiang S Yang Y Gupta S Veneziano D Athavale S González MC. The TimeGeo modeling framework for urban mobility without travel surveys. Proc Nat Acad Sci USA. (2016) 113:E5370–8. 10.1073/pnas.1524261113 27573826 PMC5027456

[31] Toole JL Colak S Sturt B Alexander LP Evsukoff A González MC. The path most traveled: travel demand estimation using big data resources. Transp Res C Emerg Technol. (2015) 58:162–77. 10.1016/j.trc.2015.04.022

[32] Klein B LaRock T McCabe S Torres L Privitera F Lake B. Assessing changes in commuting and individual mobility in major metropolitan areas in the United States during the COVID-19 outbreak. In: Northeastern University Network Science Institute. Boston, MA (2020).

[33] Legeby A Koch D Duarte F Heine C Benson T Fugiglando U. New urban habits in Stockholm following COVID-19. Urban Studies. (2022). 10.1177/00420980211070677 PMC10230291 37273493

[34] Liu Z Zhang A Yao Y Shi W Huang X Shen X. Analysis of the performance and robustness of methods to detect base locations of individuals with geo-tagged social media data. Int J Geograph Inf Sci. (2021) 35:609–27. 10.1080/13658816.2020.1847288

[35] Navaratnam AM Shrotri M Nguyen V Braithwaite I Beale S Byrne TE. Nucleocapsid and spike antibody responses post virologically confirmed SARS-CoV-2 infection: an observational analysis in the Virus Watch community cohort. Int J Infect Dis. (2022) 123:104–11. 10.1101/2022.02.01.22270269 35987470 PMC9385348

[36] Beale S Patel P Rodger A Braithwaite I Byrne T Fong WLE. Occupation, work-related contact and SARS-CoV-2 anti-nucleocapsid serological status: findings from the Virus Watch prospective cohort study. Occup Environ Med. (2022) 1–7. 10.1101/2021.05.13.21257161 35450951 PMC9072780

[37] Hariharan R Toyama K. Project Lachesis: parsing and modeling location histories. In: International Conference on Geographic Information Science. Berlin: Springer (2004). p. 106–24.

[38] Zheng Y. Trajectory data mining: an overview. ACM Trans Intell Syst Technol. (2015) 6:1–41. 10.1145/2743025

[39] Zhao K Tarkoma S Liu S Vo H. Urban human mobility data mining: an overview. In: 2016 IEEE International Conference on Big Data (Big Data). Washington, DC: IEEE (2016). p. 1911–20.

[40] Csáji BC Browet A Traag VA Delvenne JC Huens E Van Dooren P. Exploring the mobility of mobile phone users. Physica A. (2013) 392:1459–73. 10.1016/j.physa.2012.11.040

[41] Phithakkitnukoon S Smoreda Z Olivier P. Socio-geography of human mobility: a study using longitudinal mobile phone data. PLoS ONE. (2012) 7:e39253. 10.1371/journal.pone.0039253 22761748 PMC3386290

[42] Liu Z Shi W Zhang A. Detecting home countries of social media users with machine-learned ranking approach: a case study in Hong Kong. Appl Geograp. (2021) 134:102532. 10.1016/j.apgeog.2021.102532

[43] Lucchini L Centellegher S Pappalardo L Gallotti R Privitera F Lepri B. Living in a pandemic: changes in mobility routines, social activity and adherence to COVID-19 protective measures. Sci Rep. (2021) 11:1–12. 10.1038/s41598-021-04139-1 34961773 PMC8712525

[44] Ahuja N. Dot pattern processing using Voronoi neighborhoods. IEEE Trans Pattern Anal Mach Intell. (1982) 3:336–43. 10.1109/TPAMI.1982.4767255 21869045

[45] Miles R Maillardet R. The basic structures of Voronoi and generalized Voronoi polygons. J Appl Probab. (1982) 19:97–111. 10.2307/3213553

[46] Longley PA Goodchild MF Maguire DJ Rhind DW. Geographic Information Systems and Science. John Wiley & Sons (2005).

[47] Canzian L Musolesi M. Trajectories of depression: unobtrusive monitoring of depressive states by means of smartphone mobility traces analysis. In: Proceedings of the 2015 ACM International Joint Conference on Pervasive and Ubiquitous Computing. (2015). p. 1293–304.

[48] Williams NE Thomas TA Dunbar M Eagle N Dobra A. Measures of human mobility using mobile phone records enhanced with GIS data. PLoS ONE. (2015) 10:e0133630. 10.1371/journal.pone.0133630 26192322 PMC4507852

[49] Lu X Bengtsson L Holme P. Predictability of population displacement after the 2010 Haiti earthquake. Proc Nat Acad Sci USA. (2012) 109:11576–81. 10.1073/pnas.1203882109 22711804 PMC3406871

[50] Gonzalez MC Hidalgo CA Barabasi AL. Understanding individual human mobility patterns. Nature. (2008) 453:779–82. 10.1038/nature06958 18528393

[51] Kruskal WH Wallis WA. Use of ranks in one-criterion variance analysis. J Am Stat Assoc. (1952) 47:583–621. 10.1080/01621459.1952.10483441

