# Supplementary material for: Human mobility variations in response to restriction policies during the COVID-19 pandemic: An analysis from the Virus Watch community cohort in England, UK
Source: Front Public Health. 2022 Oct 14;10:999521. doi: 10.3389/fpubh.2022.999521 (PMC9623896; doi:10.3389/fpubh.2022.999521)
Supplement: Supplementary file 1 [file Data_Sheet_1.pdf]

Supplementary Material

1 SUPPLEMENTARY TABLES AND FIGURES

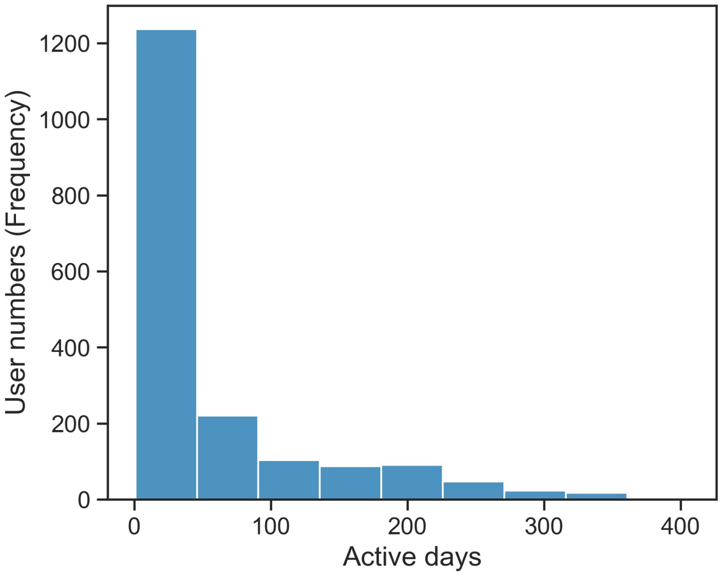

Figure S1. The distribution of active days of all Virus Watch users.

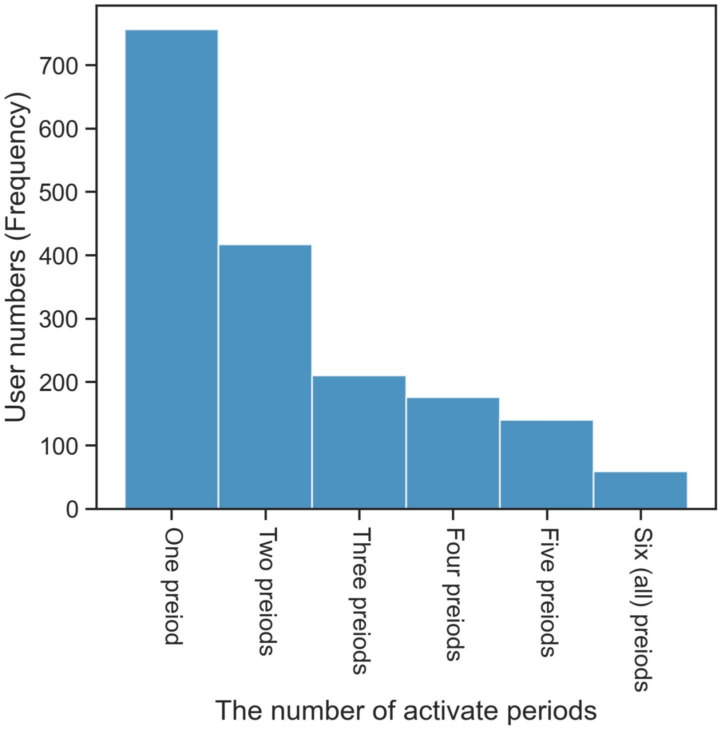

Figure S2. The distribution of active periods of all Virus Watch users.

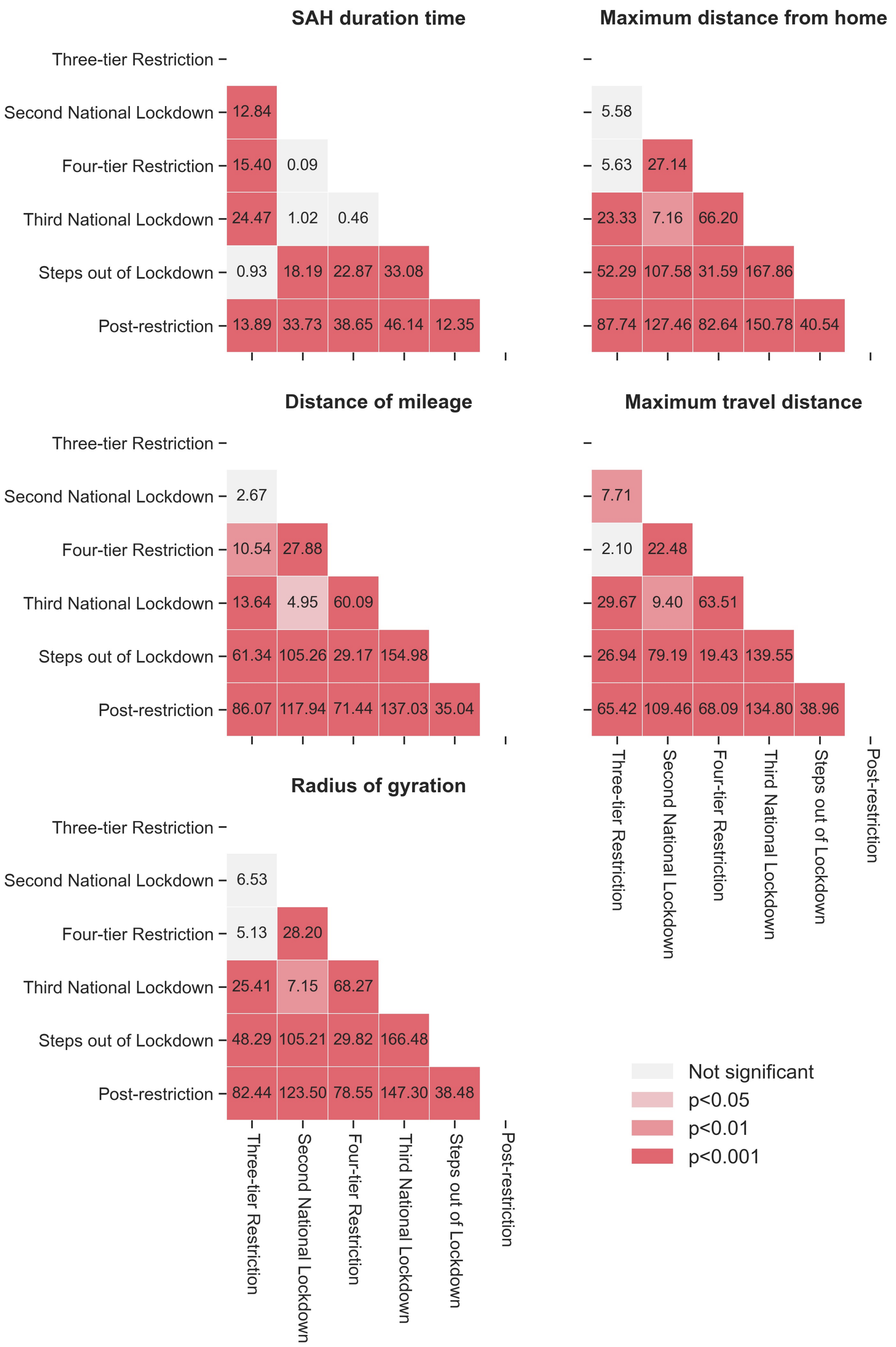

Figure S3. The differences of stay-at-home duration time and distance-based travel indicators between each restriction period. The statistic of the KWH test is plotted at each cell, and the red colour (with three types in saturation) denotes the p-value level.

Table S1. The number of detected visit/stay at places in six restriction periods. the restriction periods are 'Three-tier Restriction(TTR)'; 'Second National Lockdown(SNL)'; 'Four-tier Restriction(FTR)'; 'Third National Lockdown(TNL)'; 'Steps out of Lockdown(SOL)'; 'Post-restriction(PR)'. Place types are 'Accommodation, eating and drinking'(AED), 'Transport'(TRA), 'Commercial services'(CS), 'Attractions'(ATT), 'Sport and entertainment'(SE), 'Education and health'(EH), 'Public infrastructure'(PI), 'Manufacturing and production'(MP),'Retail'(RET) and others.

	TTR	SNL	FTR	TNL	SOL	PR	Total
**AED**	3,041	5,137	7,712	9,245	19,488	3,722	48,345
**TRA**	7,819	14,502	17,069	32,428	53,937	11,320	137,075
**CS**	11,981	24,815	29,836	56,713	81,533	13,441	218,319
**ATT**	1,733	3,372	4,396	8,479	12,837	2,272	33,089
**SE**	4,197	10,419	11,559	23,160	30,742	5,176	85,253
**EH**	6,853	15,560	20,011	37,007	52,568	8,237	140,236
**PI**	1,036	1,371	1,976	3,693	7,923	977	16,976
**MP**	1,347	2,783	4,049	7,692	9,450	1,710	27,031
**RET**	3,771	6,842	9,282	12,244	26,688	4,692	63,519
**Others**	38,876	81,832	97,130	188,949	258,827	43,622	709,236
**All stay numbers**	80,654	166,633	203,020	379,610	553,993	95,169	1,479,079

Table S2. Stay-at-home and travel indicators (mean value) in gender groups during the whole observation period.

	Gender groups		
**Human mobility indicators**	**Male**	**Female**	**KWH test**
Maximum distance from home (km)	6.52	6.92	0.01
Distance of mileage (km)	14.39	14.15	0.01
Maximum travel distance (km)	6.65	6.73	0.08
Radius of gyration (km)	2.56	2.71	0.01
Stay-at-home duration time (hour)	3.95	3.83	1.18

Table S3. Stay-at-home and travel indicators (mean value) in age groups during the whole observation period.

	Age groups				
**Human mobility indicators**	**<35**	**35 – 49**	**50 – 64**	**>64**	**KWH Test**
Maximum distance from home (km)	6.95	7.89	7.61	5.24	25.94***
Distance of mileage (km)	18.41	15.81	16.2	11.01	34.32***
Maximum travel distance (km)	6.36	7.54	7.69	5.41	24.66***
Radius of gyration (km)	2.73	3.12	2.99	2.05	24.46***
Stay-at-home duration time (hour)	3.98	4.07	3.88	3.86	0.78

*** $p < 0.001$

Table S4. Stay-at-home and travel indicators (mean value) in income groups during the whole observation period.

	Income groups				
**Human mobility indicators**	**0 – 24,999**	**25,000 – 49,999**	**50,000 – 74,999**	**>74,999**	**KWH Test**
Maximum distance from home (km)	5.5	6.77	7.75	7.01	26.48***
Distance of mileage (km)	12.71	13.61	16.73	15.33	27.95***
Maximum travel distance (km)	5.6	6.76	7.92	7.32	22.59***
Radius of gyration (km)	2.18	2.62	3.05	2.79	24.62***
Stay at home duration time (hour)	4.09	3.98	3.74	3.66	5.17

*** $p < 0.001$

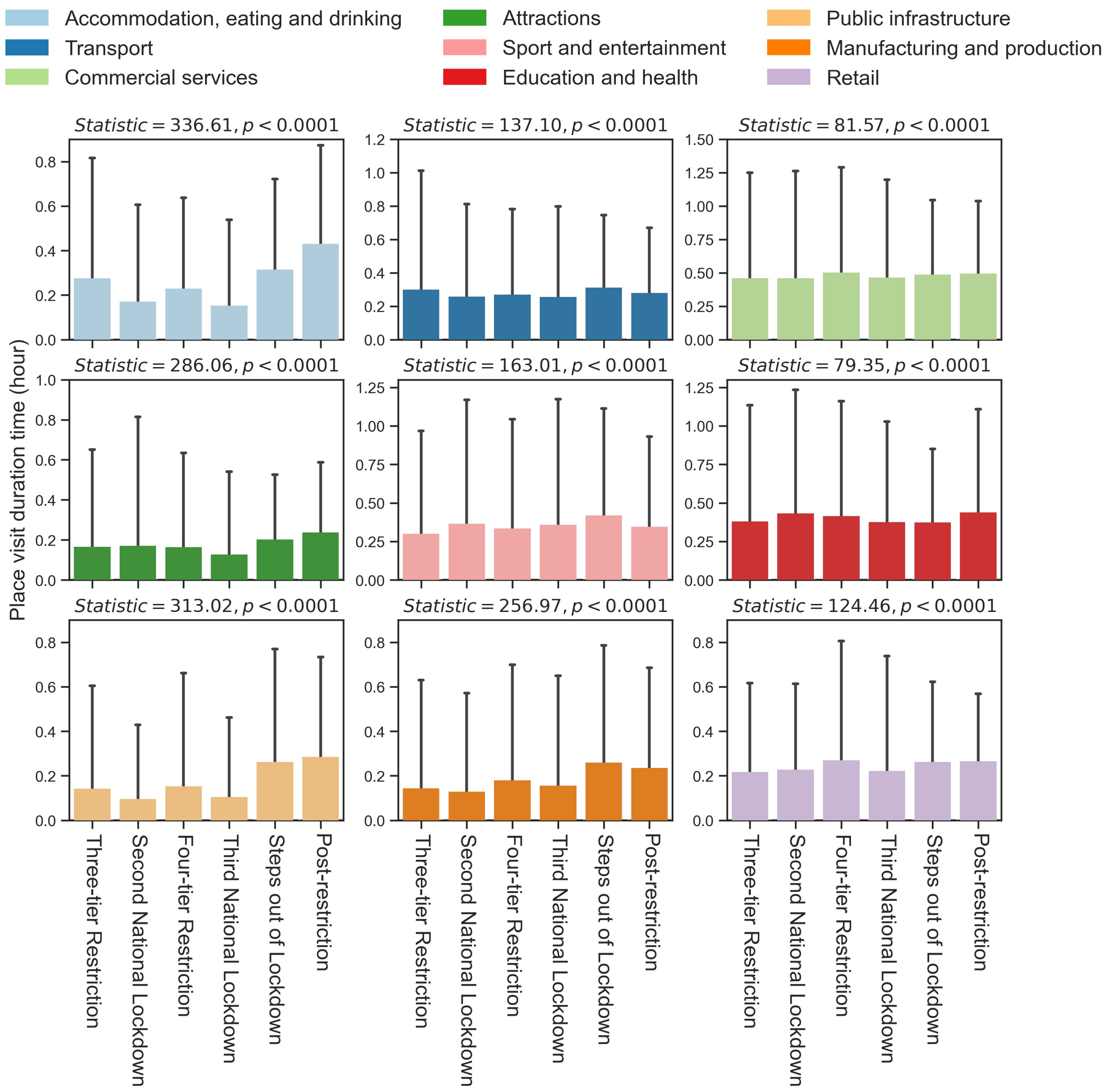

Figure S4. Place visit duration time (mean and standard deviation) at nine types of places in the six restriction periods.

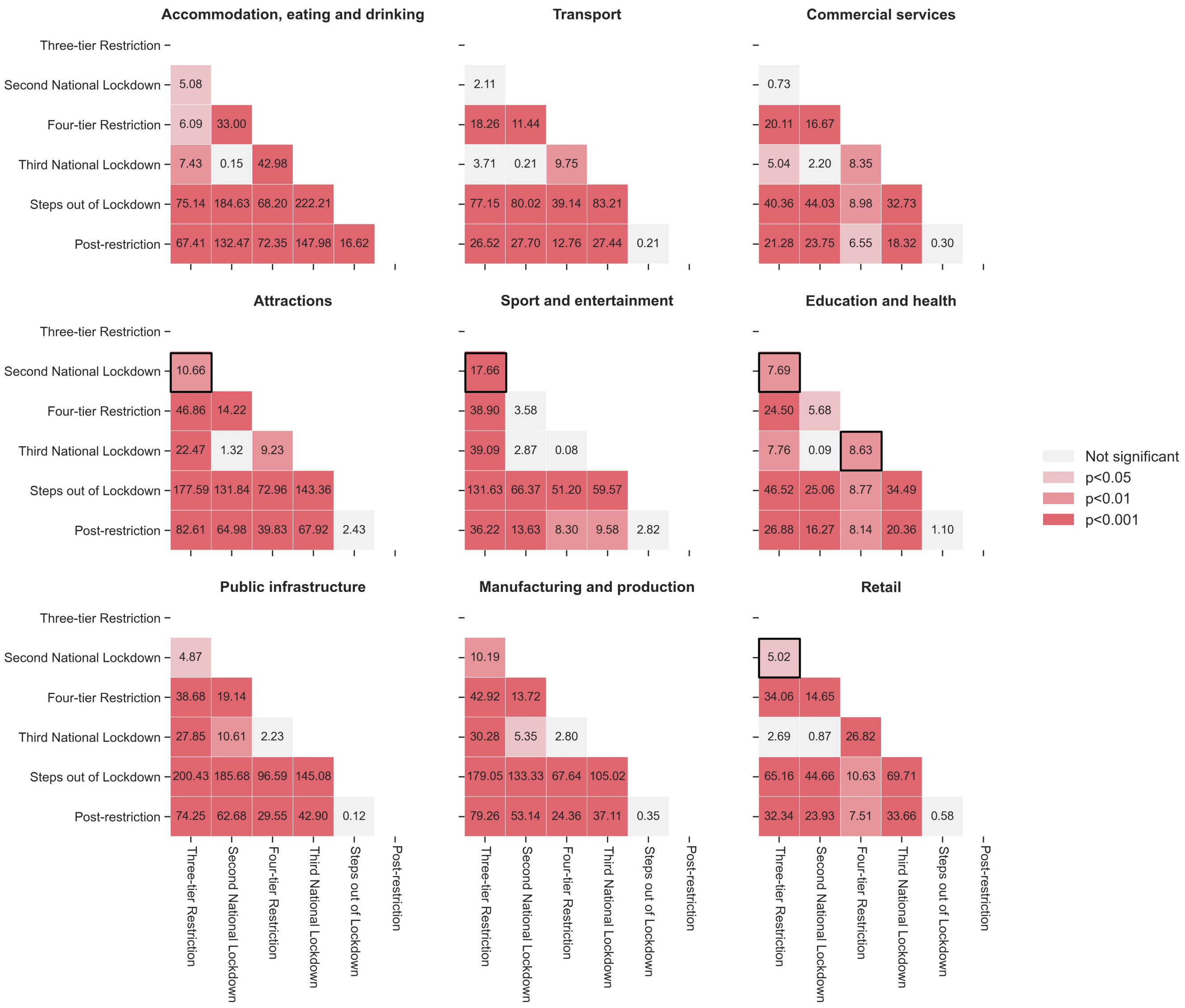

Figure S5. The paired KWH tests of place visit duration times between restriction periods. The grids with black boxes correspond to the boxes in Figure 4 of Section 4.1.

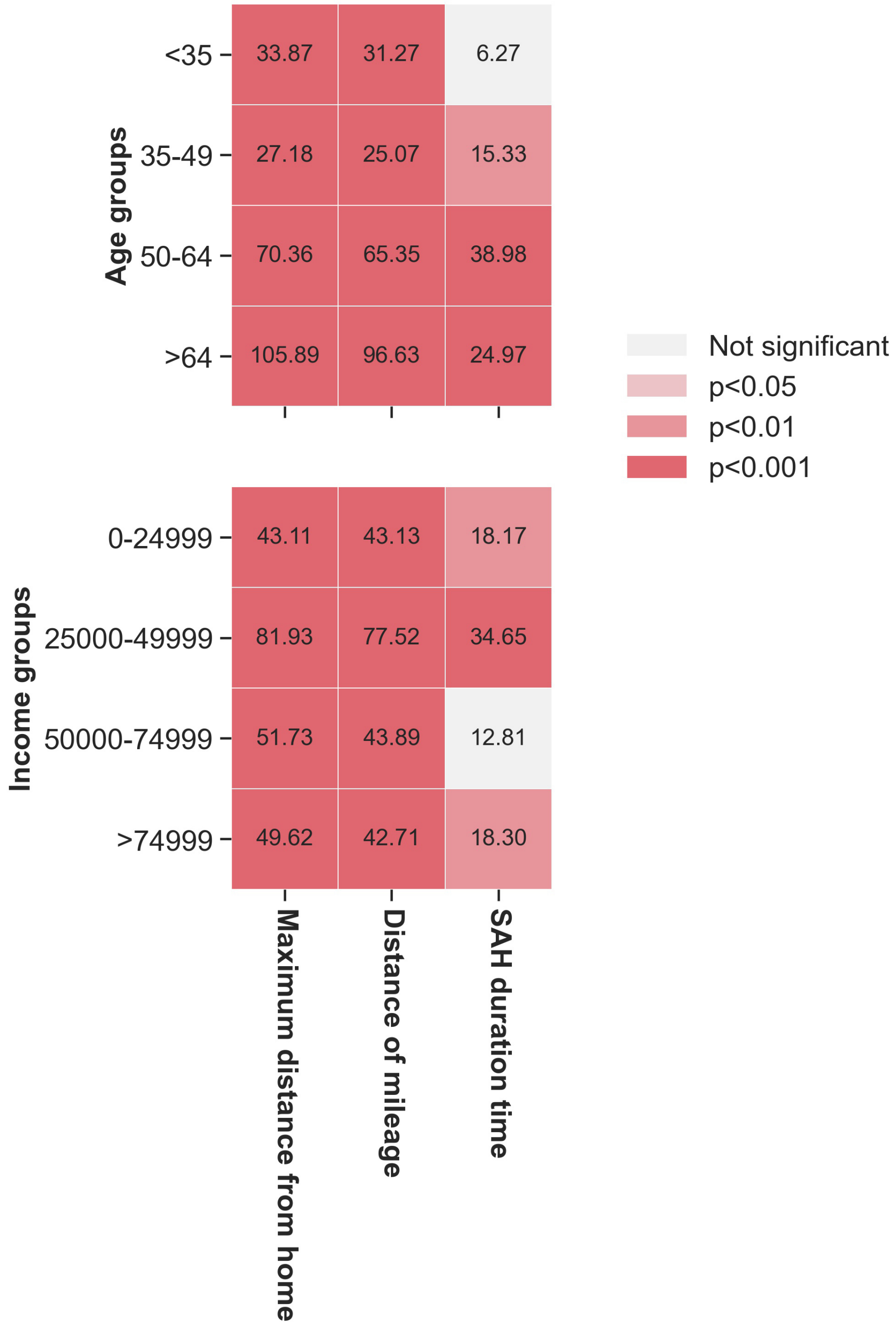

Figure S6. The KWH tests of mobility indicators in all restriction periods. Each grid with red colour denotes the indicators are significantly different during all periods.

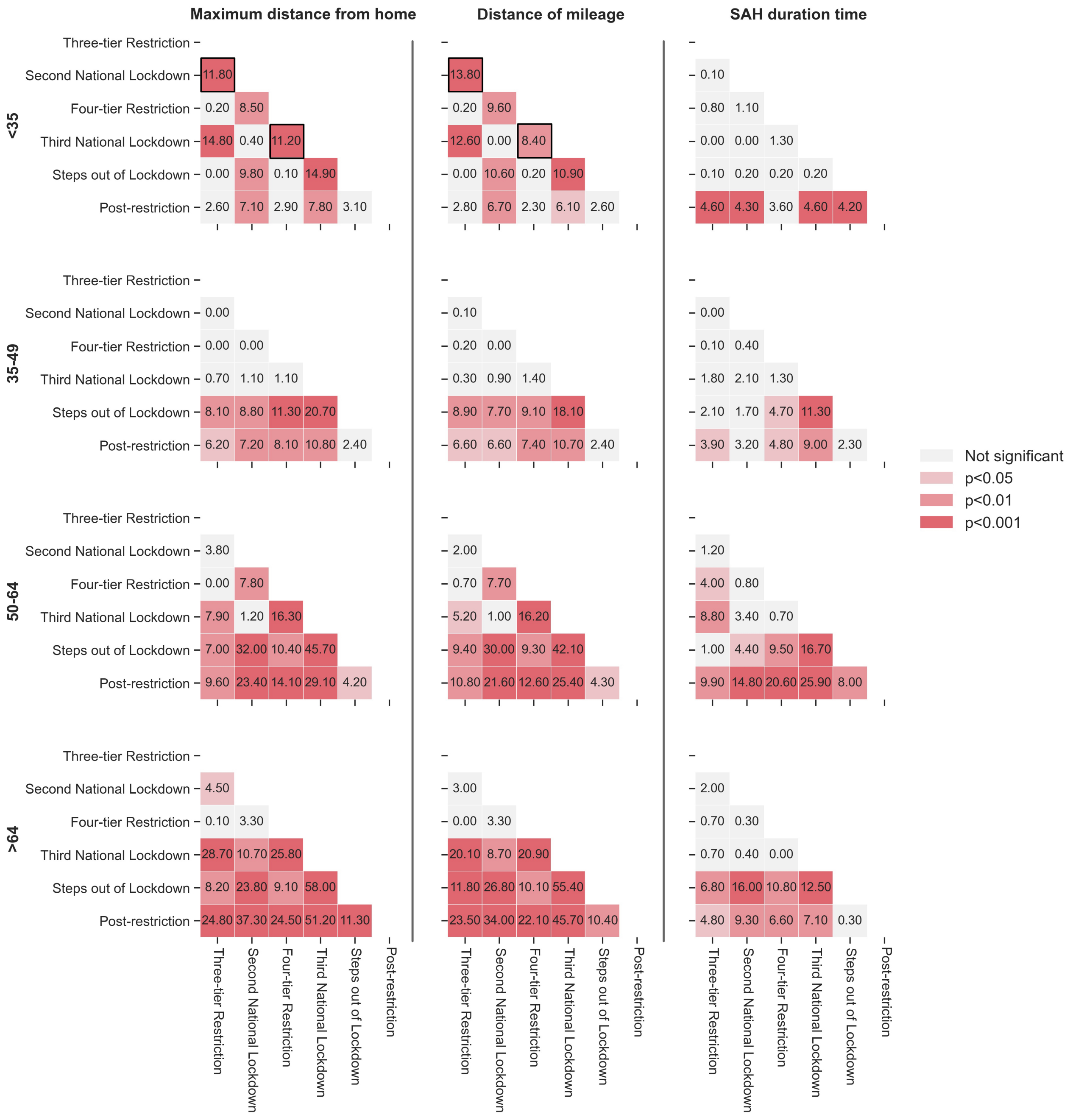

Figure S7. The paired KWH tests of mobility indicators by age groups between every two restriction periods. The grids with black boxes correspond o the boxes in Figure 5 of Section 4.2.

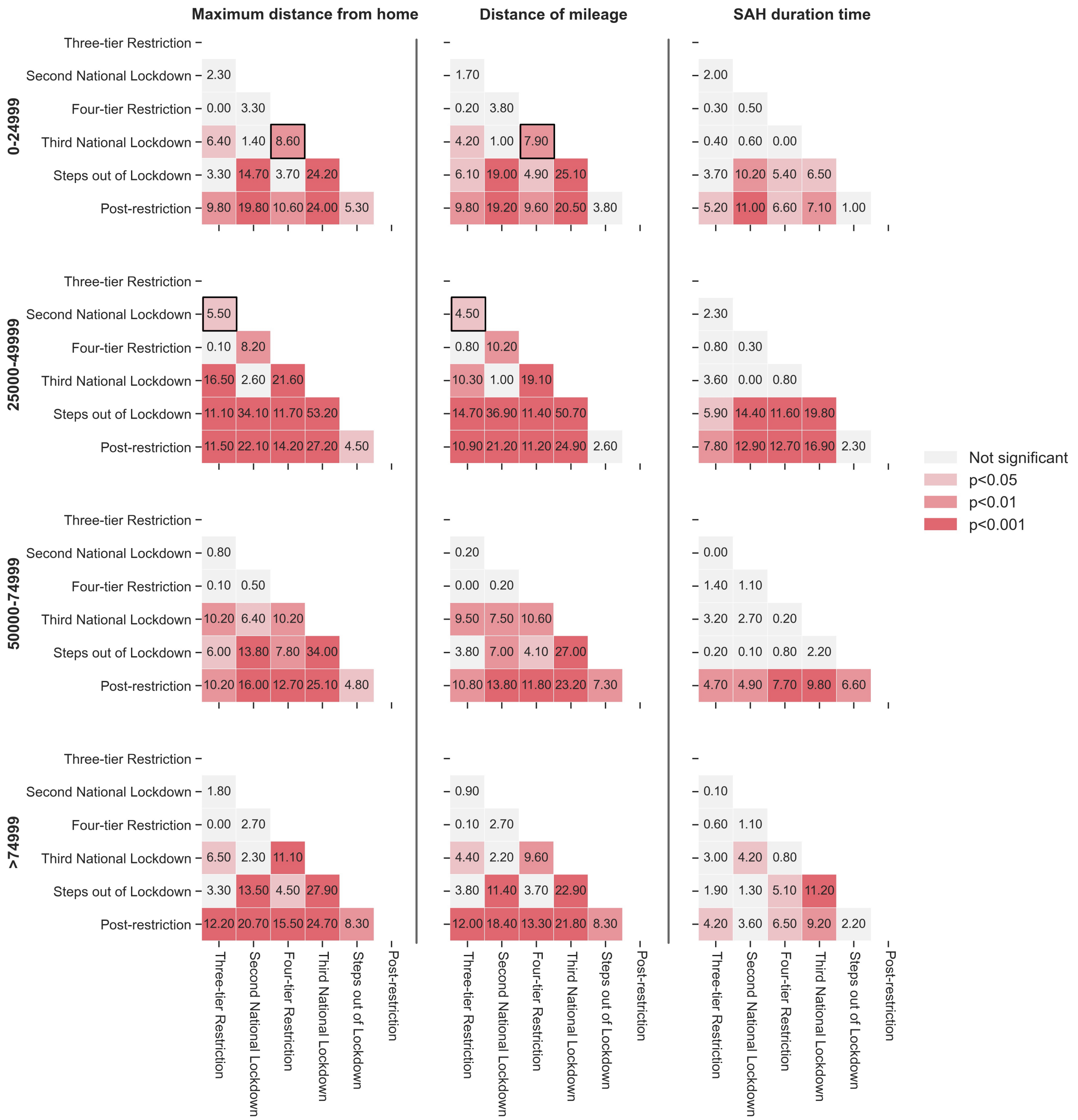

Figure S8. The paired KWH tests of mobility indicators by income groups between every two restriction periods. The grids with black boxes correspond o the boxes in Figure 5 of Section 4.2.
